# Karatekas educated on injury prevention and supported by fitness coaches are more likely to practise injury prevention

**DOI:** 10.5114/biolsport.2023.112089

**Published:** 2022-02-18

**Authors:** Montassar Tabben, Dušana Augustovičová, Jeremy Coquart, Khalid Alkhelaifi, Karim Chamari, Rafael Arriaza

**Affiliations:** 1 Aspetar Qatar Orthopaedic and Sports Medicine Hospital, Doha, Qatar; 2 Faculty of Physical Education and Sports, Comenius University, Bratislava, Slovakia; 3 Univ. Lille, Univ. Artois, Univ. Littoral Côte d’Opale, ULR 7369 – URePSSS – Unité de Recherche Pluridisciplinaire Sport Santé Société, F-59000 Lille, France; 4 Physical Education and Sports School, Universidade da Coruña, A Coruña, Spain; 5 Instituto Médico Arriaza y Asociados, A Coruña, Spain

**Keywords:** Combat sports, Prevention, Epidemiology, Implementation, Perception

## Abstract

To determine the current perceptions and practices of top-level karate athletes concerning risk factors and injury prevention programme (IPP) implementation in training and competition. Out of 90 eligible countries (933 athletes) participating in the karate World Senior Championships (WSC) in Madrid 2018, 50 countries (55.6%) represented by 137 athletes (14.7%; 52 females and 85 males) responded to a structured questionnaire. Of the athletes responding, 45% reported that their national team did not conduct any measures to reduce injury risk (43% among females and 47% among males; p = 0.68). Kumite athletes (51%) were more likely to practise injury prevention compared to kata athletes (25%; p = 0.016). Of the respondents, 69%, 60%, 60% and 34% reported having no team doctor, fitness coach, massage therapist and physiotherapist, respectively. A greater proportion of athletes who had access to a fitness coach (part-time or full-time) engaged in injury prevention strategies (67% and 51%, respectively) than those who did not (35%; p = 0.031). Athletes who had received previous advice about injury prevention were more likely to practise injury prevention (58%) compared to the rest (21%; p < 0.001). The current study revealed that: i) almost half of the karatekas already benefited from an injury prevention programme, ii) injury prevention programmes were practised more frequently when there was a fitness coach among their coaching staff, iii) karatekas who had received education about injury prevention were more likely to practise injury prevention programmes.

## INTRODUCTION

In competition, prospective studies have shown that karate athletes sustain on average one injury every 25 min of combat, although the large majority of these are minor or mild in severity [1]. This remarkable injury rate, equivalent to 2400 injuries per 1000 competition hours, exists despite efforts by the World Karate Federation (WKF) to mitigate risk of injury by regularly revising the rules of the game to protect the health of the karateka [2]. In training, very few studies, using survey data, have shown that training related injuries represent 76 to 90% of all injuries [3, 4]. Indeed, competitions are just the end process of a karate athlete’s preparation and do not account for exposure during training and sparring combat. Studying only competition injuries therefore does not provide a complete measure of injury risk, and could lead to selection and survivorship bias [5], because non-selected and/or injured athletes who could not participate in competitions are simply ignored. Therefore, it is important to implement effective strategies to prevent injuries in both training and competition.

However, studies related to injury prevention programmes (IPPs) in karate are lacking. Recently, IPPs have gained considerable attention in many sports [6–8], as injury prevention is considered important to minimise the associated treatment costs, participation loss and long-term negative side effects for the athletes [9], In addition, scientific evidence, especially in football, has demonstrated that these programmes can significantly prevent injuries [10–13]. Before studying the efficacy of any IPP, it is important to understand the reality of IPP implementation and end-users’ perceptions toward IPP [8], including why karate athletes do, or do not, adopt IPPs. This would generate a better understating of the factors influencing successful implementation of IPPs.

Therefore, the purpose of the present study was to determine the current perceptions and practices of top-level karate athletes concerning risk factors and injury prevention programme (IPP) implementation in training and competition.

## MATERIALS AND METHODS

### Design

Cross-sectional study based on face-to-face surveys.

### Participants

A total of 1117 athletes (140 countries) participated in the karate World Senior Championships (WSC) in Madrid, 2018. Because of the language barrier, 933 athletes (90 countries) were potentially eligible to answer the available questionnaire languages (English, French, Slovak, and/or Spanish). During the pre-event official medical meeting, the Chairman of the World Karate Federation Medical Commission presented the project and invited the team doctors to inform their athletes about the face-to-face survey and ask them to present at least one athlete as a representative of the team to conduct the questionnaire. Data were collected between 6^th^ and 11^th^ November 2018 and athletes were asked to base their responses on their programmes and experiences from the past season (2017–2018). All participating athletes signed informed written consent to participate.

This study was approved by the Ethics Committee of the Faculty of Physical Education and Sports, Comenius University, Bratislava, Slovakia (reference number 13/2019).

### Survey

The survey was constructed in English, French, Slovak, and Spanish. The questionnaire consisted of 15 questions (see online supplementary appendix *Injury Prevention WKF questionnaire*) with four sections: (i) injury prevention implementation overview, (ii) perceptions and attitudes towards injury prevention, (iii) perceived injury risk and injury prevention practice by body part, and (iv) perceived injury risk factors.

The design of the survey took into consideration the authors’ combined knowledge and experience of sports medicine and sports science in elite karate. The survey was pilot tested with 20 high-level karate athletes (who did not participate in the WSC) before the start of the study. The received feedback and comments were taken into consideration and changes were made in response to these.

### Survey analysis

Raw data were entered separately by DC and LP from paper questionnaires to Microsoft Excel and then double-checked case by case by MT. The data were analysed using the SPSS 22.0 program for Mac OS X (SPSS, Inc., Chicago, IL, USA). Crosstabs using chi-square tests were used to analyse proportions.

## RESULTS

### Survey information

Out of 90 countries (933 athletes) eligible to the survey languages, 50 countries (55.6%) represented by 137 athletes (14.7%; 52 females and 85 males) completed the questionnaire. However, 416 athletes did not volunteer and 380 could not be reached.

### IPP overview and athletes’ perception and attitude toward injury prevention

Of the athletes responding, 45% reported that their team had not conducted any preventive measures to reduce injury risks (43% among females and 47% among males (p = 0.68). Kumite athletes (51%) were more likely to practise injury prevention compared to kata athletes (25%; p = 0.016). Among the respondents, 69%, 60%, 60% and 34% had no team doctor, fitness coach, massage therapist and physiotherapist, respectively. Athletes who had a fitness coach (part-time or full-time) employed better injury prevention strategies (67% and 51%, respectively) compared to athletes who did not have a fitness coach (35%; p = 0.031).

The availability of medical and technical staff in the teams and the relationship with injury prevention practice are presented in Table 1.

**TABLE 1:** Medical and technical staff availability in top-level karate athletes according to injury prevention implementation status

	Number of athletes who Practiced injury prevention with their team (%)	Number of athletes who never Practiced injury prevention (%)	p-value
**Team Doctor**			
No	30 (43.5)	39 (56.5)	
Yes, Part time	8 (44.4)	10 (55.6)	0.833
Yes, Full time	15 (50)	15 (50)	

**Physiotherapist**			
No	12 (35.3)	22 (64.7)	
Yes, Part time	22 (45.8)	26 (54.2)	0.283
Yes, Full time	20 (54.1)	17 (45.9)	

**Massage therapist**			
No	24 (40)	36 (60)	
Yes, Part time	13 (48.1)	14 (51.9)	0.318
Yes, Full time	17 (56.7)	13 (43.3)	

**Fitness coach**			
No	21 (35)	39 (65)	
Yes, Part time	14 (66.7)	7 (33.3)	0.031*
Yes, Full time	18 (51.4)	17 (48.6)	

* significant difference (P < 0.05).

Athletes who had received previous advice about injury prevention were more likely to practise injury prevention (58%) compared to those who had not received any advice (21%; P < 0.001). As many as 88% of the participating athletes thought that injury prevention is important in karate, while 90% of respondents had a positive or very positive attitude towards injury prevention measures.

The perception and attitude of the karate athletes toward injury prevention are presented in Table 2.

**TABLE 2:** Perception and attitude of top-level karate athletes toward injury prevention

	Number of athletes Practiced injury prevention with their team (%)	Number of athletes never Practiced injury prevention (%)	*p*-value
**Have you previously received advice about injury prevention programs**			
Yes	40 (58)	29 (42)	P < 0.001*
No	9 (20.5)	38 (79.5)	

**Importance of injury prevention**			
Low or Moderate	7 (47)	8 (53)	0.841
High	47 (44)	60 (56)	

**It is more important to use the training time to play karate than to conduct injury prevention**			
Fully agree or Agree	34 (50)	34 (50)	
Disagree or Fully disagree	12 (36)	21 (64)	0.356
Not sure	8 (38)	13 (62)	

**Attitude towards injury prevention measures**			
Positive or Very Positive	49 (45)	60 (55)	0.548
Neutral or Negative or very negative	4 (33)	8 (67)	

* significant difference (P < 0.05).

### Perceived injury risk and ‘importance’ of injury prevention according to body parts

**FIG. 1:**
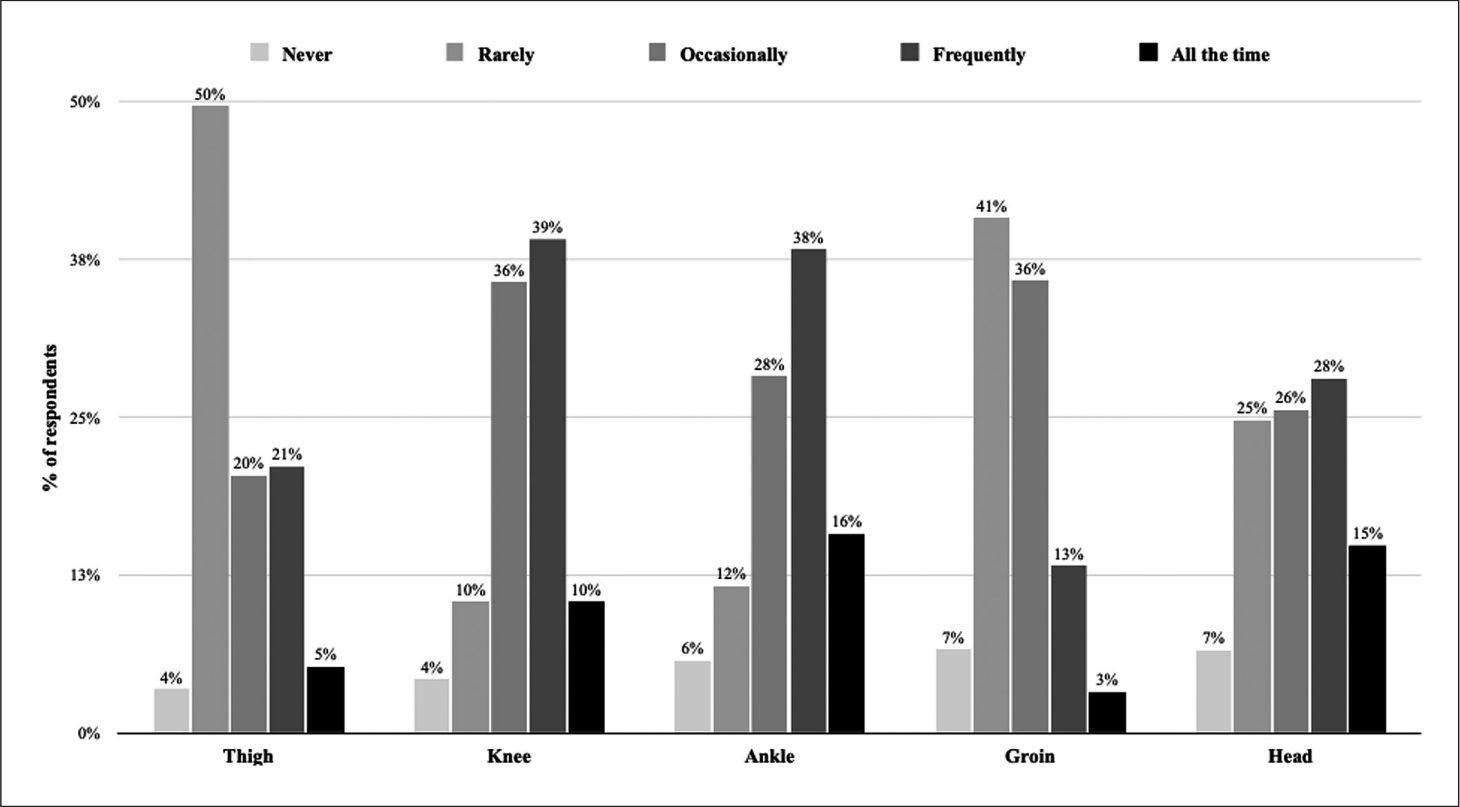
Injury risk perception related to injury body part.

Of the surveyed athletes, 54%, 50% and 43% responded that the ankle, knee, and head were the most frequently (frequently or all the time) injured body parts. Their perceived importance of injury risk according to body part is detailed in Figure 1.

**FIG. 2:**
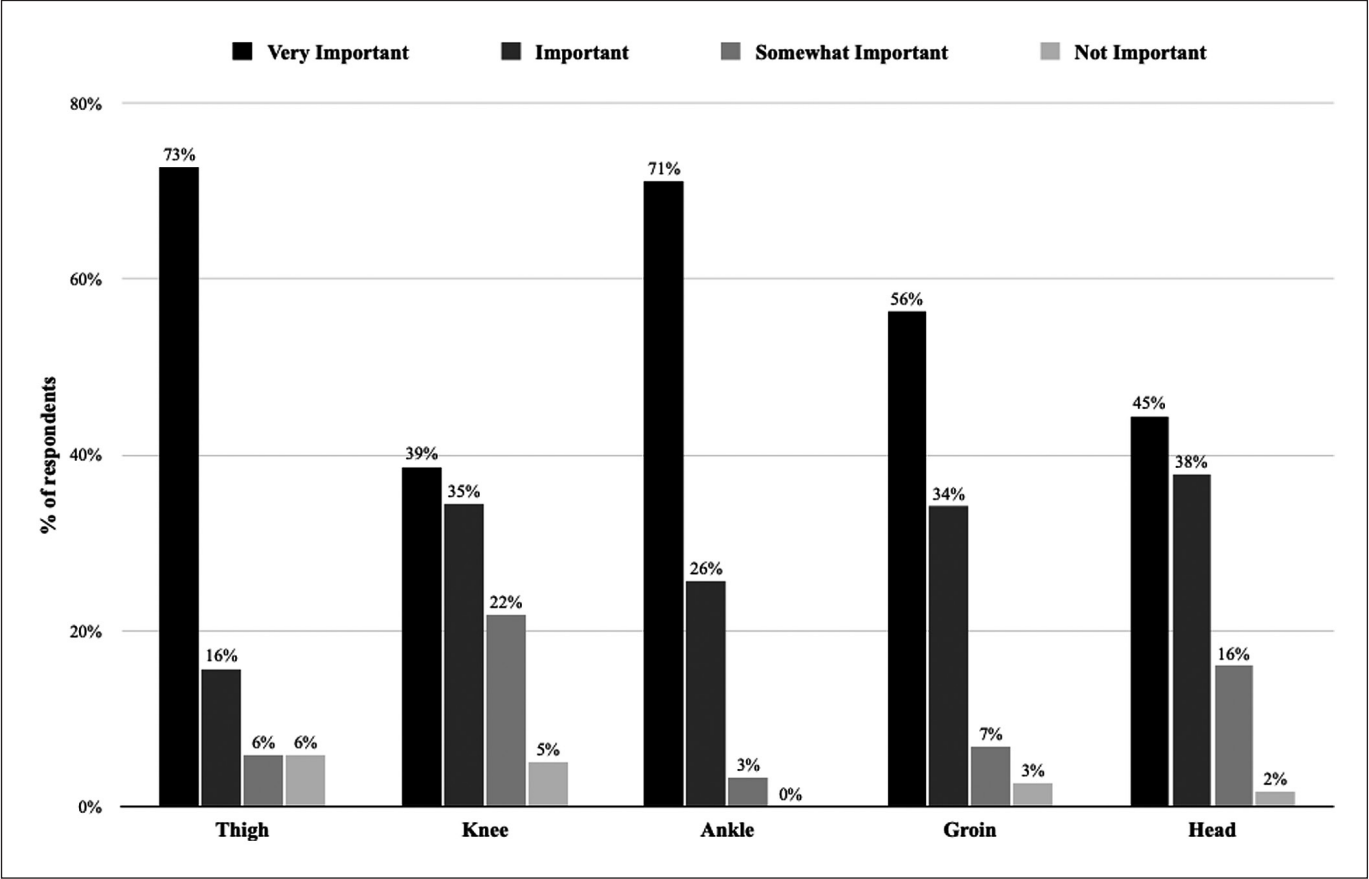
The perceived injury prevention importance by body part.

The most important body parts that karate athletes think should be protected most are the thigh, knee and ankle. Participants’ perceived ‘importance’ of injury prevention regarding body parts is presented in Figure 2.

### Perceived injury risk factors

**FIG. 3:**
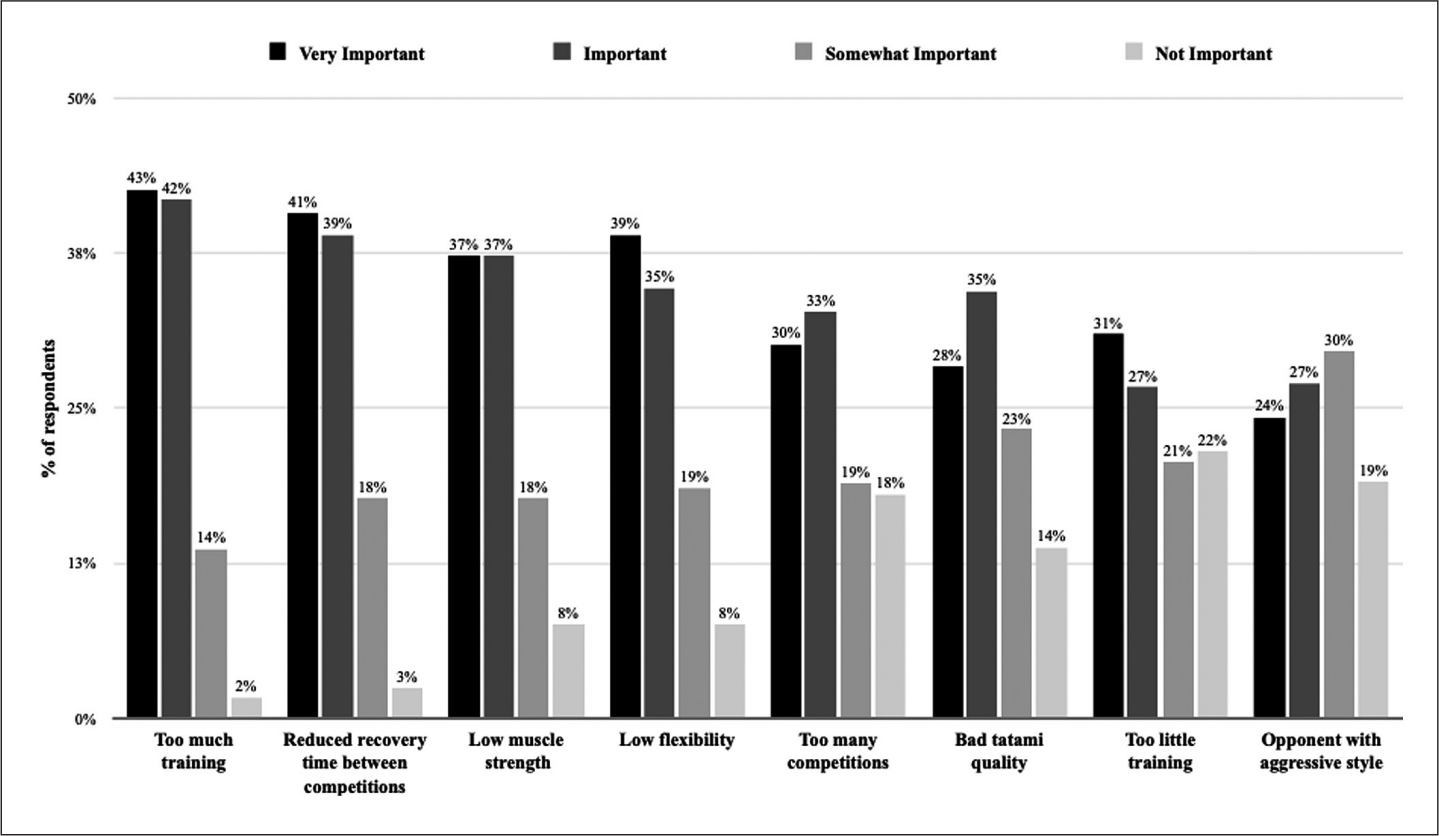
Perception of risk factors by importance

**FIG. 4:**
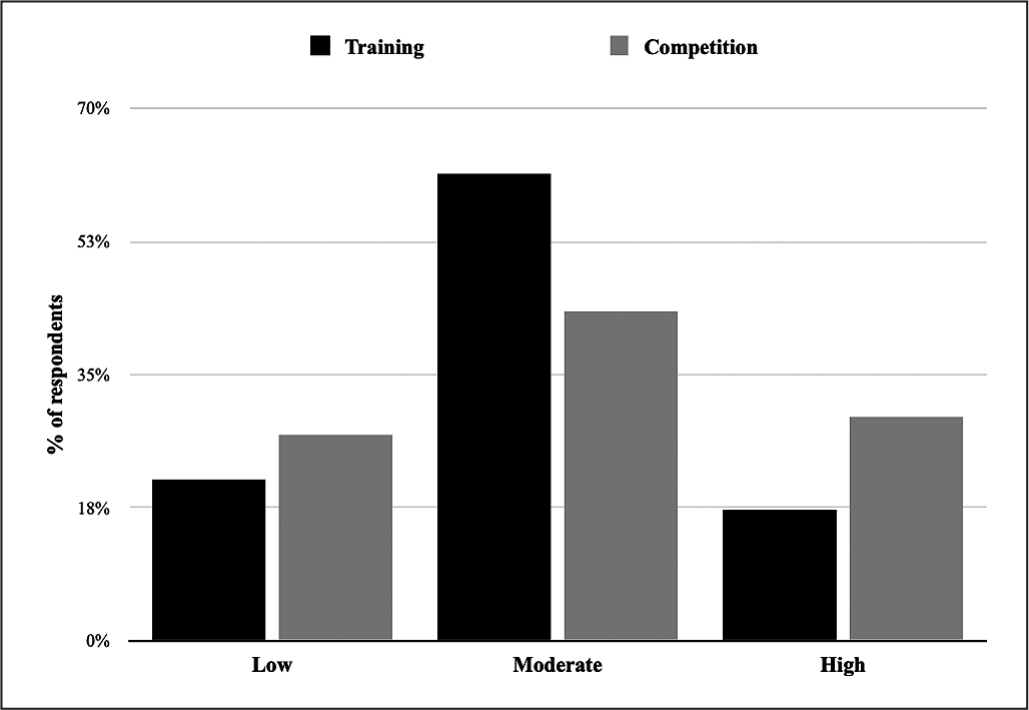
Training vs. competition injury risk perception.

The perception about injury risk in competition compared to training is presented in Figure 3. Based on perceived rating of importance by athletes, the three most important risk factors for injury are presented in Figure 4.

## DISCUSSION

This is the first study to directly observe the beliefs and considerations of IPP and provides insights into the perceptions of IPP in elite karate. The current results reveal that: i) 45% of the participating athletes reported that their team had not conducted any preventive measures to reduce injury risks, ii) injury prevention programmes were practised more frequently when the athletes had a fitness coach, iii) athletes who had received previous advice about injury prevention were more likely to practise injury prevention programmes, and iv) the main risk factors for injuries were thought to be ‘too much training’, ‘lack of recovery time between competitions’, and ‘low muscle strength’. The findings of this study will help inform practitioners about the barriers and facilitators of IPP in elite karate.

### IPP overview and athletes’ perception and attitude toward injury prevention

In the present study, nearly half of the participating athletes reported that they had not conducted any preventive measures to reduce injury risks. Much of the research on IPP so far has focused on team sports, with player adherence identified as a key factor for IPP success [12]. However, the results of this study indicated that this low implementation rate could not be linked to players’ adherence, as almost all of the respondents reported that an injury prevention programme is important to reduce injury occurrence and were positive or very positive towards injury prevention measures.

Whilst athletes are the intended health beneficiaries of IPP, team staff also play a key role in achieving the desired injury prevention [14]. In the present study, most respondents were not supported by medical staff – there was no team doctor, fitness coach, massage therapist or physiotherapist. Consequently, the low implementation rate could be linked to the availability of the individuals delivering programmes within the team. Indeed, there was a greater proportion of athletes who had a fitness coach (part-time or full-time) than those without one. Two-thirds of respondents reported having a physiotherapist available in their team. Our findings reinforce previous studies from professional football, emphasizing the key role of fitness coaches and physiotherapists in the implementation of IPP. O’Brien et al. [15] found that in football, fitness coaches were responsible for delivering the IPPs, while physiotherapists assisted with the supervision and correction of exercises.

The current study also shows that player education is a key factor and major point of divergence when it comes to practising compared to never practising injury prevention. Indeed, respondents who had received previous advice about injury prevention were nearly three times more likely to practise IPP compared to karatekas who had not received any advice. This aligns closely with the fact that education and communication of scientific evidence play an important role in convincing players about injury prevention benefits [8, 12]. The findings suggest that team managers should ensure the availability of at least part-time fitness coaches and physiotherapists within their teams.

### Perceived ‘importance’ of injury prevention according to body parts

A recent systematic review and meta-analysis on injury epidemiology in WKF karate indicated that the most commonly injured body parts were the head and neck, and the lower limbs [1]. Despite the fact that the 28 studies included in this meta-analysis were all from competitions (e.g. championships and tournaments), a similar distribution was reported by our respondents, with ankle, knee and head/face injuries perceived as the most frequent (without differentiating between training and competition). Interestingly, in the current study, athletes did not clearly distinguish between body parts when asked about their prevention priorities. It seems that karatekas have a relatively accurate picture of the injury epidemiology, yet do not make any clear differentiation when it comes to preventing injuries. For them, all body parts seem nearly equally important. This is an interesting observation and may simply reflect that injury to any small body part (e.g. a broken finger) could stop the athlete from practising karate. Therefore, this is a point of reflection for clinicians. Should we focus on the most frequent/serious injuries for prevention, and ignore the rare ones, or should we consider any injury as a potential threat to the athletes’ career and therefore consider prevention more broadly?

### Perceived injury risk factors

The main perceived injury risk factors in the current study were: ‘too high training load’, ‘too short recovery period between competitions’, ‘lack of muscle strength’ and ‘lack of flexibility’. This is consistent with Destombe et al. [3], who previously suggested that ‘more time spent training each week’ was associated with an increased risk of injury in karate. On the other hand, recent studies in football have indicated that lack of recovery between matches and high training load were perceived by the practitioners as the two main key extrinsic risk factors for injury [12, 13]. Therefore, the monitoring of training load could appear as crucial in preventing injuries among elite karatekas. In karate, several methods using competition/training duration and heart rate or rating of perceived exertion to quantify the training load have previously been validated and could be a useful support for karate practitioners [16–18]. Nevertheless, this issue needs further investigations to verify whether the perception of the athletes turns out to be correct, i.e. that a high training load is actually associated with an increased risk of injuries.

‘Lack of muscle strength and flexibility’ was considered, in the current study, as a major perceived injury risk factor in karate. This aligns with the few studies focusing on IPP showing that muscle strength and flexibility were frequently assessed from screening tests in elite soccer to prevent non-contact injuries [12, 13, 19]. However, to date, no studies have documented an effect of flexibility training as prevention, and this finding in the present study refers to karate athletes’ view. Indeed, this could be the nature of karate – with greater demands on flexibility than running sports or soccer. It would therefore be interesting to test the effects of an injury prevention programme including both eccentric resistance and flexibility exercises in elite karate.

Arriaza et al. [2] reported that the global injury incidence during World Karate Championships was almost double with the old rules compared to the one with the new rules. This was a consequence of a WKF long-term strategy to mitigate the risk of injury during competition. We speculate that the WKF initiative aiming mainly at competitions derived from the fact that scores concerning injuries came mostly from studies that focused on competition, whereas few studies deal with injuries sustained during training [20]. However, the results of the present study confirmed that karate athletes believe they are at the same risk of sustaining injuries in both training and competition. This aligns with previous studies showing the great risk during karate training and reporting that training-related injuries represented between 76% and 90% of total annual injuries in karate [3, 4]. The challenges and opportunities arising from this study need careful consideration by the WKF to reinforce their current strategies of reducing injuries by focusing on IPP during training to prepare for competitions.

### Limitations and considerations

There are some limitations to our survey. First, there is a gap concerning the level of some teams/countries, in which resources (e.g., equipment and staff numbers) will vary and will influence the knowledge, attitude and practices of teams. Second, only 137 karatekas participated in this survey (14.7% of all the athletes taking part in the competition). However, the sample represented 50 countries (55.6%), which could provide a general picture of the injury prevention situation in the presented countries. This response rate could be explained by various reasons: (1) gaining access to elite karatekas for face-to-face interview during karate WSC is challenging, (2) the language barrier, as only athletes who spoke English, French, Slovak, Polish, Czech and/or Spanish took part in this study, (3) some athletes deemed the nature of the information to be too sensitive to disclose.

This study provides knowledge about how karatekas perceive the injury prevention programmes. However, this knowledge and good intentions to use it do not guarantee adoption.[21] Key considerations are how these programmes will be delivered and by whom. As noted by O’Brien and Finch (7) and O’Brien and Donaldson (21), it will be essential to address all levels of the system when developing karate injury prevention programmes and their related implementation plans to identify potential barriers (e.g., lack of knowledge, time or programme acceptance) and facilitators to programme adoption.

## CONCLUSIONS

The current study revealed that: i) almost half of the karatekas have already benefited from an injury prevention programme, ii) injury prevention programmes are more frequently practised when a fitness coach is present in the athlete’s coaching staff, iii) karatekas who had received education about injury prevention were more likely to practise injury prevention programmes.
